# Immunomodulatory, inflammatory, functional and biomechanical benefits of chronic exercise in physically active vs. insufficiently active older after fourth COVID-19 vaccine dose: nine months cohort

**DOI:** 10.1016/j.clinsp.2026.101014

**Published:** 2026-06-26

**Authors:** Lucas Silva Mantovanelli, Tatiane Silva de Souza, Ellen Cristina Pertinhez, André Luis Lacerda Bachi, Rita Santos-Rocha, Ana Paula Ribeiro

**Affiliations:** a Health Science Post-Graduate Department, Medicine School, Biomechanics and Musculoskeletal Rehabilitation Laboratory, Universidade Santo Amaro, São Paulo, SP, Brazil; b Technical University of Lisbon, Laboratory of Biomechanics and Functional Morphology, Neuromechanics Research Group of Human Movement, CIPER, Lisboa, Portugal and Sport Sciences School of Rio Maior, Polytechnic Institute of Santarém, Portugal; c Universidade de São Paulo, Physical Therapy Department, School of Medicine, São Paulo, SP, Brazil

**Keywords:** Older, Exercise, Inflammation, Immunological, Vaccine, Function, Gait

## Abstract

**Introduction:**

The vaccination against COVID-19 is being carried out, and observing the aging process and changes in the face of the pandemic in the older adults, studies are needed to better understand these changes. The purpose of this study was to compare immune response, inflammation, physical-functional performance, and gait at one and nine months after the fourth dose of the COVID-19 vaccine between physically active and insufficiently active older adults.

**Methods:**

Prospective cohort study. Thirty older adults were evaluated and divided into two groups: the Experimental Group ‒ physically active (EG, *n* = 15) and the Control Group ‒ insufficiently active (CG, *n* = 15). Assessments were baseline, one and nine months, post-vaccination and regular exercise practice. Main Outcome Measures: Immunological and inflammatory markers and physical-functional variables: questionnaires (FRAQ, Baecke), physical tests (Step Test, Floor Transfer Test, Timed Up and Go, six-minute walk test, hand grip strength), and gait analysis using a pressure platform.

**Results:**

The EG showed increased IgA and decreased IgG levels over time. Regarding inflammation, EG demonstrated modulation of the inflammatory response, characterized by increased IL-10 levels, significant changes in IL-12p70 and IP-10 concentrations, and improved pro-/anti-inflammatory cytokine ratios (e.g., IL-6/IL-10), indicating a more regulated immune profile. Additionally, EG improved physical performance in the Step Test, Floor Transfer Test, and six-minute walking test, along with reduced plantar load in the midfoot and rearfoot regions. In contrast, the CG showed reductions in IL-10 and IP-10 levels over time, without consistent improvement in inflammatory modulation or physical-functional performance. In inter-group comparisons at nine months, EG exhibited higher IL-10 levels and a more favorable pro-/anti-inflammatory balance compared with CG, suggesting a controlled and effective immune activation following vaccination.

**Conclusion:**

The practice of regular physical exercise was associated with more favorable immune, inflammatory, physical-functional, and gait outcomes at one and nine months after the fourth dose of the COVID-19 vaccine in physically active older adults compared with insufficiently active peers. These findings suggest that long-term regular physical exercise was associated with modulation of the immune-inflammatory profile, marked by a balanced increase in both Th1-associated cytokines (IL-12p70, IP-10) and the regulatory cytokine IL-10 and increased IgA levels and a reduction in IgG over time, suggesting a controlled and effective immune activation following vaccination against COVID-19 and regular exercise practice compared with CG (insufficiently active) and better physical-functional performance with reduction plantar overloading during gait.

## Highlights

• Active older adults: ↑IgA, ↓IgG; IL-10, IL-12p70, IP-10 modulated.
• Regular exercise improved inflammatory balance: ↑IL-10, ↓IL-6/IL-10 over 9-months.
• Active group improved physical-functional performance and gait load at 9-months.
• Inactive adults: ↓IL-10, IP-10; no consistent immune/functional gains.
• Regular exercise improved immunity, inflammation balance, function and gait.

## Introduction

The older adult population has been growing exponentially, with estimates indicating about 1.2 billion people over 60-years of age by 2025 and 1.4 billion by 2030.^1^^,^^2^ Senescence is a dynamic and progressive process that impairs physical and functional capacity through the gradual decline in muscle mass and strength ‒ characteristic of sarcopenia ‒ along with balance loss, increasing vulnerability to falls, functional limitations, and sedentary behavior.^3^^,^^4^ These factors culminate in high economic costs with medical and hospital expenses for patients, family members, and policy management bodies in health care for older.^5^^,^^6^ In addition to the changes inherent to the senescence process, the world has faced the COVID-19 pandemic, caused by the novel coronavirus SARS-CoV-2, which has had major economic, public health, and mental health impacts, especially among older adults.^7^^,^^8^ In Brazil, the first confirmed case was reported on February 26, 2020, and within five months, 98.3% of municipalities had registered infections .^8^, ^9^, ^10^

In December 2019, a new coronavirus was identified as causing flu-like illness and serious pulmonary complications, Coronavirus Disease 2019 (COVID-19) .^11^ The incubation period for the virus is an average of five days and can vary from two to 14 days. Most infected adults (≈90%) present mild symptoms; however, older adults and those with comorbidities ‒ such as cardiovascular or pulmonary disease, diabetes, and hypertension ‒ may develop severe conditions, including respiratory failure and death, with fatality rates between 2%–5% .^12^^,^^13^

To combat COVID-19, governments implemented measures such as isolation, social distancing, and educational campaigns emphasizing hygiene and mask use .^14^^,^^15^ Health services, in turn, faced significant challenges in providing safe and effective care to patients affected by COVID-19 .^16^, ^17^, ^18^, ^19^ Although necessary, these measures affected social dynamics^6^^,^^19^ and contributed to increased sedentary behavior and reduced physical activity levels ‒ one of the main adverse effects of social isolation .^20^, ^21^, ^22^ Among older adults, this decline in physical activity intensified motor impairments and accelerated the effects of senescence .^2^^,^^6^^,^^23^^,^^24^

Systematic reviews have shown that individuals over 60-years are the most affected by COVID-19, with high mortality risk due to age-related immune decline leading to severe respiratory infections ^25^^,^^26^, and reduced physical-functional performance .^25^^,^^27^^,^^28^ COVID-19 also impacts the musculoskeletal system through inflammatory imbalance ‒ marked by increased pro-inflammatory and reduced anti-inflammatory cytokines^4^ ‒ along with loss of contractile proteins, reduced mitochondrial density, and replacement of muscle tissue by fat and connective tissue .^29^, ^30^, ^31^ These changes cause weakness, fatigue, impaired gait, and balance deficits, increasing vulnerability to falls .^30^^,^^31^ Sarcopenia and balance loss further contribute to immobility, early institutionalization, and higher morbidity and mortality among older adults .^32^, ^33^, ^34^

One of the main clinical strategies to combat COVID-19 and its effects on older adults' health was vaccination, whose safety and efficacy became a global priority .^35^ Most vaccines target the viral spike glycoprotein, responsible for binding to the ACE2 receptor and enabling viral entry .^35^ Vaccines such as ChAdOx1 nCoV-19 (AstraZeneca/Oxford), JNJ-78,436,735 (Janssen), BNT162b2 (Pfizer/BioNTech), and CoronaVac (Butantan/Sinovac) were approved for emergency use .^35^^,^^36^ An effective immune response requires balanced inflammatory modulation and antibody production, ensuring the formation of immune memory.

Worldwide, mass vaccination programs have been developed based on the principle that prevention is more effective and less costly than treating chronic diseases, aiming to reduce morbidity and mortality .^36^ Considering the effects of aging and the impact of COVID-19 on the immune, inflammatory, and physical-functional systems of older adults ^25^^,^^29^, ^30^, ^31^, ^32^, ^33^, along with increased sedentary behavior due to social isolation[23,24] further studies are needed to clarify immune and inflammatory responses after the 4th vaccine dose and the return to regular chronic exercise. In this context, it is essential to investigate cytomegalovirus reactivation, COVID-19 antigen response, and inflammatory modulation before and after vaccination, as well as to assess physical-functional performance and gait biomechanics over nine months post-exercise resumption, to support targeted rehabilitation strategies for older adults during the pandemic. Thus, the aim of the present study was to compare immune response, inflammation, physical-functional performance, and gait at one and nine months after the fourth dose of the COVID-19 vaccine between physically active and insufficiently active older adults.

## Methods

### Design, setting, participants, and ethics

This research is a prospective cohort study in which older people were recruited between March and December 2022, totaling nine full months of follow-up. This observational cohort study was conducted in accordance with the STROBE Statement (Strengthening the Reporting of Observational Studies in Epidemiology). A total of 30 older people, of both sexes, who were immunized against COVID-19, completing the 4th vaccination dose for the disease, were recruited for evaluation. The older adults were allocated into two groups according to their long-term pre-pandemic exercise habits:

- Experimental Group ‒ Physically Active (EG, n = 15): older adults with a history of regular physical exercise practice for at least five consecutive years prior to the pandemic, with a minimum frequency of two sessions per week;
- Control Group ‒ Insufficiently Active (CG, n = 15): older adults with no consistent long-term exercise history prior to the pandemic, reporting either sedentary behavior or irregular practice (≤ 1 session per week) during the year preceding the study.

It is important to emphasize that the group allocation (EG vs. CG) was determined by their exercise habits in the years leading up to the pandemic, not by their resumption of exercise afterwards. The 24-month “washout” applies to both groups, as everyone was isolated during the COVID-19 pandemic. Thus, it is important to explicitly clarify that the Experimental Group (EG) did not exercise during lockdown, and the study follows their resumption of regular exercise practice, while the Control Group (CG) remained sedentary during lockdown and their activity level remained stable and did not increase during the study period.

Assessments took place at three moments: 1) Pre-vaccination of COVID-19; 2) One month after vaccination of COVID-19 and regular exercise practice; and 3) 9 months after vaccination of COVID-19 and regular exercise practice. It is worth mentioning that the physical-functional performance variables were only evaluated at moments 1 and 3.

This study was previously submitted to the Research Ethics Committee of the Universidade Santo Amaro ‒ UNISA, obtaining approval under opinion number: 5.418.231. All older women who participated in the research previously signed the free and informed consent form, drawn up in accordance with resolution 466/12 of the National Health Council. Data were collected at the Biomechanics and Musculoskeletal Rehabilitation Laboratory of the University.

The eligibility criteria for this study were: older people between 60- and 80-years of age, immunized with the 4th dose of vaccination against COVID-19 (Janssen or Pfizer vaccines), a body mass index less than 35 kg/m^2^; Sleep quality self-reported as normal, habitual diet without nutritional supplementation, without uncontrolled vestibulo-cochlear diseases, cardiac and/or respiratory arrhythmias, convulsive syndrome, musculoskeletal or neurological disorders, diabetic neuropathies, and tissue lesions (tegumentary ulcers of any etiology) that are functionally limiting. They were also required not to have prostheses and/or orthoses of the lower limbs or fractures in the previous 3-months, not to present signs of dementia, and be able to walk independently, that is, demonstrate a good general state of health.

### Immunological and inflammatory assessment protocol

Fasting peripheral blood samples were collected up to five days before and after 30-days of administration of the 4th dose of vaccine for COVID-19 in appropriate tubes to obtain aliquots of serum (minimum of 500 μL) from blood clotting in the collection tube itself and subsequent centrifugation at 2500 rpm for 10-minutes at 4 °C, which were subsequently frozen at −80 °C for further analysis .^37^

### Determination of antibody concentrations for SARS-CoV-2

The systemic concentration of antibodies of the IgA, IgM, and IgG isotypes specific to SARS-CoV-2 antigens was determined in serum samples using the “in-house” ELISA technique, adapting the protocol described by Bachi et al .^37^ Briefly, the 96 wells of the microplate were coated with nCoV-PS-Ag7 antigens and subsequently blocked with PBS-BSA-T buffer containing 1% bovine serum albumin in PBS (1 ×, pH = 7.3) +0.05% Tween 20. After washing three times with PBS-T solution (PBS 1 ×, pH = 7.3 + 0.05% Tween), the serum [diluted 1:2000 for IgA and IgM or 1:10,000 for IgG in PBS-BSA (PBS 1 ×, pH = 7.3 + 0.1% BSA) was added and incubated for 2-hours at 37 °C. After three washes with PBS-T solution, peroxidase-conjugated secondary antibody diluted 1:5000 for IgA and IgM or 1:10,000 for IgG in PBS-BSA was added. After incubation for 1 h at 37 °C and three washes with PBS-T, 100 μL of the TMB solution (3,3′.5,5′-tetramethylbenzidine) was added and incubated for 10-minutes at room temperature, avoiding direct exposure to light. The reaction was stopped by adding 50 μL of a sulfuric acid solution (0.2 N) to each well, and the optical density was read at 450 nm .^37^

### Determination of cytokine concentrations

The concentrations of the pro-inflammatory profile cytokines (IFN-α2, IFN-β, IFN-λ1, IFN-λ2/3, IFN-γ, IL-1β, IL-6, IL-8, IL-12p70, IP-10), and TNF-α, as well as the anti-inflammatory IL-10, were determined in serum samples by a multiplex assay (LEGENDplex™, Biolegend, San Diego, CA, USA). In this sense, all serum samples were initially diluted 2-times, and then 25 μL of the diluted sample was used to perform this assay. The concentration of each cytokine was calculated using appropriate standard curves. The linearity of the multiplex assay was within the range of 2.4 to 10,000 pg/mL, which includes the range of sample determinations. The correlation coefficients of all standard curves ranged from 0.95 to 0.99, while the intra-assay coefficients of variance were 3% to 5% and the inter-assay coefficients of variance were 8% to 10%. The analysis was performed with the BD Accuri™ C6 Plus flow cytometer (BD Biosciences San Jose. CA. USA), and the data obtained were analyzed with LEGENDPlex™ V8.0 software (Biolegend) .^37^

### Physical-functional performance assessment protocol

The physical-functional assessments were only evaluated at two moments: before vaccination against COVID-19 and nine months after vaccination against COVID-19 and regular exercise practice. These assessments consisted of the application of questionnaires: FRAQ-Brazil (Falls Risk Awareness Questionnaire) to assess the perception of risk of falls in the older adults, and a modified Baecke questionnaire to assess the level of physical activity. In addition, physical tests were applied: the Step Test to evaluate the performance of going up and down stairs and the floor transfer test to evaluate physical performance. To assess walking, the six-minute walk test was applied, and for dynamic balance, the dynamic balance test using the Timed Up & Go Test (TUG) .^38^

The FRAQ-Brazil questionnaire was used to assess the perception of fall risk in individuals over 65-years of age. The questionnaire consists of 25 multiple-choice questions, in which the total score varies from 0 (minimum score) to 32 (maximum score), with the higher the score, the better the perception of fall risks .^38^^,^^39^

The modified Baecke habitual physical activity questionnaire for older adults (QBMI) was used to investigate physical activity in the previous 12-months in the older adults. The questionnaire consists of 16 questions, divided into three domains: 1) Activities of daily living; 2) Physical exercise or sporting activity practiced during leisure time; and 3) Physical activities during leisure time and physical mobility activities. The calculation is performed using information about sports and other leisure-time activities, in relation to the type of activity, duration (hours per week), frequency (number of months per year), and intensity with which the activity was normally carried out .^40^

The Step Test was used to assess physical performance. The test consists of placing one foot at a time on a 20 cm high stable wooden support base and then repeatedly returning to the floor as quickly as possible. Participants were able to go up and down at the speed they desired in order to perform the maximum number of steps they were able to during 6 min. They could also start the test with any lower limb and change it at any time during the test; thus, the test was more like an activity of daily living .^41^

The Timed Floor Transfer (FTT) test was applied with the older adults moving from the bipedal position in the standing posture (initial position) to sitting on a mat positioned on the floor and then getting up as quickly as possible to return to the initial position. The assessment criteria were speed, agility, reaction time, muscular strength, motor coordination, sensory system (visual and somatosensory), and static balance .^42^

The six-minute walk test was used to assess the maximum distance (cm) that the patient could walk in six minutes (ATS, 2002). The test assesses the patient's ability to move during this period. All participants were instructed to walk as quickly and far as possible during the six-minute period .^41^^,^^43^

The TUG was used to verify physical performance during walking and dynamic balance. The TUG test consists of measuring the time spent in the task of getting up from a chair, walking three meters to a marker on the ground, turning around and walking back along the same route, sitting down again with the back supported on the back of the chair. To classify the test, time values between 11- and 20-seconds are considered normal for frail older adults or disabled patients. Values greater than or equal to 20 seconds indicate impairment in physical performance and balance, requiring an appropriate intervention .^41^^,^^44^

### Handgrip strength evaluation protocol

To assess muscle strength in the upper limbs, the handgrip test was performed. The older adults remained seated in a chair with the forearm supported in a neutral position and the elbow at an angle of 90° The participants performed a total of three repeated contractions in each arm, with a contraction duration of three seconds, and the repetitions were performed consecutively with the right hand and then with the left hand. Values below 30 and 20 (kg) for men and women are classified as low muscle strength .^38^

### Biomechanical assessment protocol during gait

Biomechanical assessments were evaluated only at two moments: before vaccination against COVID-19 and nine months after vaccination against COVID-19 and regular exercise practice. For biomechanical evaluation of plantar pressure distribution during gait, a pressure platform (Loran® Sensor Medica Inc., Rome, Italy) was used, with dimensions of 3240 mm long, 620 mm wide, 20 mm high, and weighing 29 kg. Part of the equipment is made up of resistive pressure sensors, homogeneously distributed (4 sensors/cm^2^). The platform was connected to a desktop notebook to transmit the data, collected at a frequency of 100 Hz .^45^

**Fig. 1:**
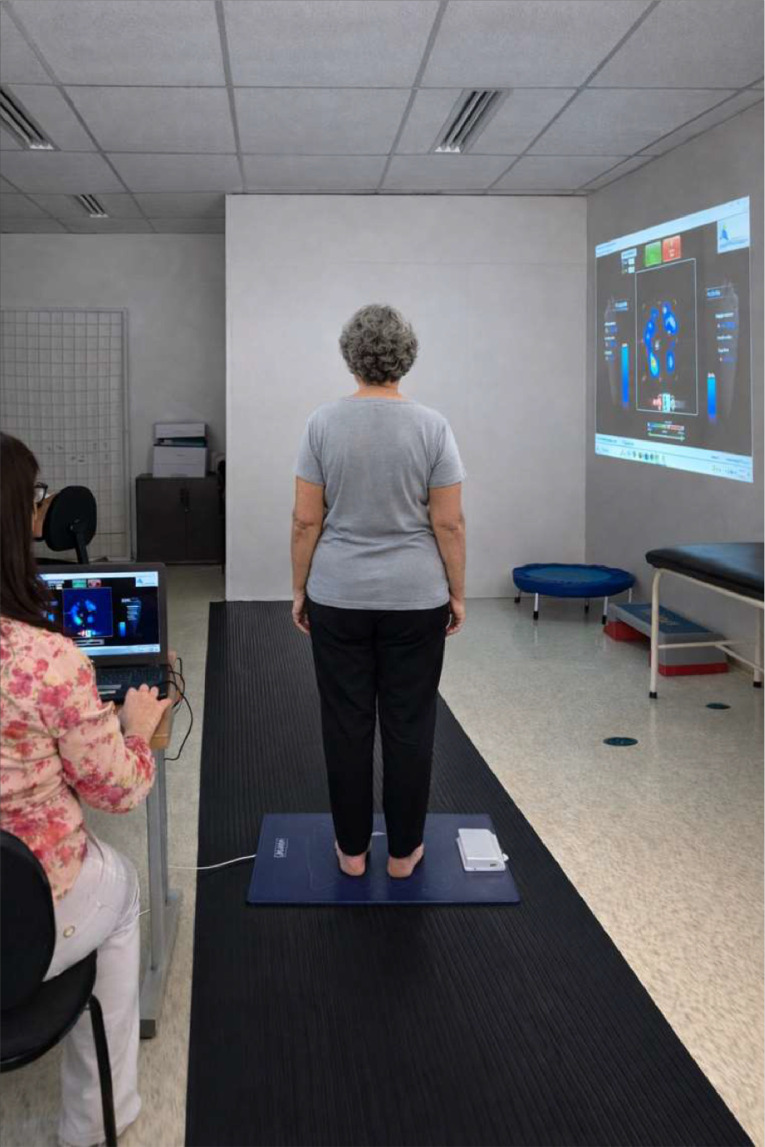
Demonstration of the analysis of plantar pressure distribution during gait of older adults evaluated in both groups (EG ‒ Experimental Group ‒ Physically Active and CG ‒ Control Group – Insufficiently Active) after one and nine months of the fourth COVID-19 vaccine dose and regular exercise practice.

The older adults walked at a pre-established cadence. To ensure that they had reached this cadence, plantar pressure acquisitions were monitored using a stopwatch. The older adults were familiarized with the collection environment and instruments to reduce the retroactive effect. After familiarization, the older adults walked on a flat synthetic rubber track at a distance of 20 meters. The steps included in the intermediate 10 meters were timed and considered valid for analysis, thus totaling approximately 12 steps, captured in 6 rounds of walking with the feet supported on the platform (Fig. 1) .^45^ The plantar pressure variables analyzed and measured were: 1) Maximum peak pressure value per selected area: represents the maximum pressure value (expressed in kPa); 2) Maximum Mean Pressure: represents the mean value of the maximum pressure (expressed in kPa); and 3) Contact area: represents the area in which the sensors were activated (pressed) in each step (expressed in cm^2^). All plantar pressure variables were analyzed in 4 plantar areas of the feet. To do this, the foot was divided into four areas: medial and lateral hindfoot (30% of foot length), midfoot (30% of foot length), and forefoot and toes (40% of foot length) .^45^

### Regular exercise training program

The continuous supervised exercise training program was carried out on the premises of the Centro Educacional e Esportivo Ibirapuera, belonging to SEME, and supervised by the same physical education professional, who monitored all stages of physical exercise progression, in order to guarantee the continuity and assiduity of the participants in the training program (Supplementary Table S1). Before the COVID-19 pandemic, all older adults were already undergoing the same training program .^37^

The supervised exercise training program was interrupted for approximately 24-months during the COVID-19 lockdown “washout period”. After restrictions were lifted, participants resumed participation under direct supervision by qualified exercise professionals, following a structured, combined training protocol including both aerobic and resistance components. Adherence was continuously monitored through attendance records, and only participants who attended at least 80% of the supervised sessions were included in the final analysis (Supplementary Table S1).

All parameters of frequency, intensity, and periodization of physical exercise were based on the recommendations of the American College of Sports Medicine and the American Heart Association. Thus, the program consisted of aerobic exercises (varying between 60% and 75% maximum heart rate) associated with localized muscular resistance exercises (bodybuilding), varying between 50% and 60% of 1RM (maximum repetition). Each aerobic training session consisted of 30-minutes of moderate-intensity aerobic exercise, ranging from 60% to 75% of maximum heart rate (FC-max), assessed using a heart rate monitor (Polar brand, model FT1, Polar ‒ Finland). A moderate intensity was maintained based on the equation 220-minus age (220-age) credited to Karvonen, with values compared to the equation 208-minus 0.7-times age (208 - 0.7 × age). Training with localized resistance exercises was carried out at moderate intensity, ranging between 50% and 60% of 1RM (maximum repetition). In total, 5 to 10 different exercises are involved for the following muscle groups: lower and upper limbs, abdomen, glutes, and those related to postural stabilization, including dorsal and lumbar. The exercises were performed in 2 series of 10 to 20 repetitions for 30-minutes a day, using weight plates or free weights immediately after the end of the aerobic training .^37^ (Supplementary Table S1).

### Statistical analysis

The total number of older people (*n* = 30) evaluated was determined through sample calculation using the G*Power program, considering a power of effect of 80%, calculated based on the equation between functionality (TUG) and gait (peak plantar pressure). Data multivariate normality was tested, and multicollinearity was ruled out prior to the MANOVA. Comparative time effects (intra-group analyses) were performed using one-way repeated-measures MANOVA or MANCOVA with Bonferroni-adjusted post-hoc tests. Cohen’s *d*-test to verify the effect of the intervention. For inter-group comparisons, Student’s *t*-tests were used. For all analyses, the significance level was set at 5% (*p* < 0.05).

## Results

**Fig. 2:**
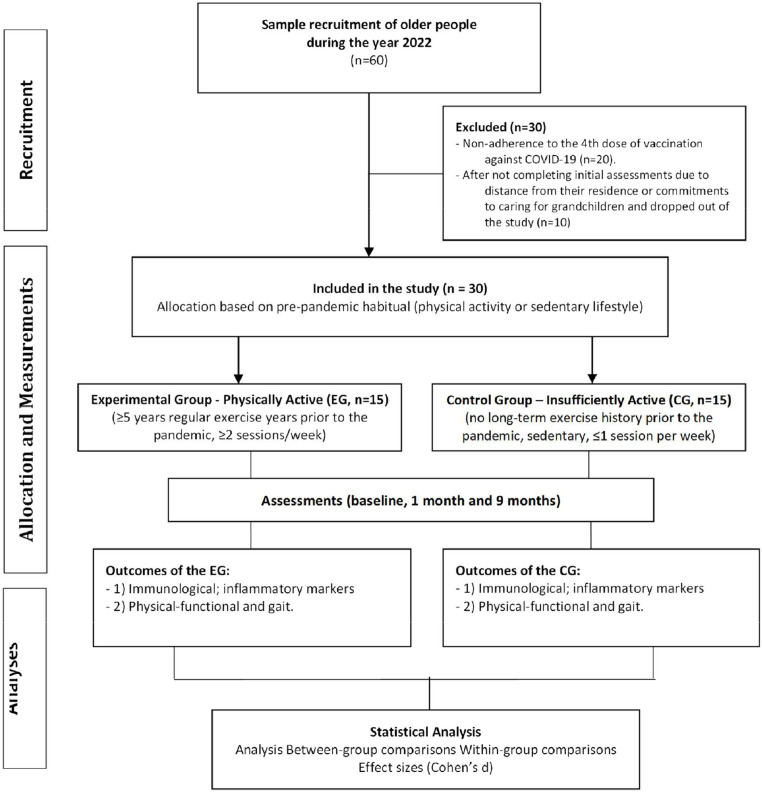
CONSORT Flow Diagram of the evaluation process of the older in baseline and after one and nine months after the fourth COVID-19 vaccine dose and regular exercise practice.

The CONSORT Flow Diagram and evaluation times were represented in Fig. 2. In Table 1, it can be observed that the older adults did not differ in anthropometric characteristics for the different assessment moments, only the time of sports practice between the groups (EG and CG).

**Table 1:** Comparison of anthropometric aspects and physical exercise between the groups: experimental group-physically active (EG) versus control group-insufficiently active (GC) after one and nine months of the fourth COVID-19 vaccine dose and regular exercise practice.

Variables	Experimental Group (EG) Physically Active					Control Group ‒ (GC) Insufficiently Active					GE × GC (baseline / 9 months)
	Baseline	1 month	9 months	*d*	p	Baseline	1 month	9 months	*d*	p^a^	p^b^
Age (years)	71.0 ± 5.5	71.1 ± 5.4	71.6 ± 5.6	0.10	0.521	75.0 ± 1.6	71.5 ± 6.3	71.8 ± 6.6	0.16	0.621	0.236
											0.627
Weight (Kg/cm^2^)	64.3 ± 13.2	64.8 ± 13.3	65.0 ± 14.1	0.05	0.438	66.2 ± 8.1	65.8 ± 13.4	65.4 ± 12.6	0.07	0.872	0.476
											0.630
Height (cm)	1.59±0.9	1.60±0.8	1.60±0.6	0.01	0.876	1.6 ± 0.8	1.58±0.6	1.58±0.6	0.02	0.149	0.653
											0.788
BMI (Kg/cm^2^)	24.0 ± 8.9	26.3 ± 5.6	26.8 ± 5.0	0.38	0.654	25.3 ± 2.6	26.4 ± 5.5	26.2.0 ± 5.4	0.21	0.609	0.532
											0.926
Physical exercise practice (years)	5.5 ± 2.5	5.5 ± 2.5	5.9 ± 2.2	0.16	0.876	1.0 ± 0.3	1.0 ± 0.3	1.0 ± 0.2	0.03	0.631	*0.010*^a^
											*0.012*^a^
Sex (F and M)	80% (F)	80% (F)	80% (F)	‒	‒	75% (F)	75% (F)	75% (F)	‒	‒	‒
	20% (M)	20% (M)	20% (M)	‒	‒	25% (M)	25% (M)	25% (M)	‒	‒	‒

BMI, Body Mass Index; F, Female; M, Male.

a MANOVA tests, significant differences *p* < 0.05. ^b^ Student *t-*test, independent, significant differences *p* < 0.05.

Table 2 shows that the older adults of the EG exhibited higher immunity to the COVID-19 virus one and 9-months after the 4th dose of the COVID-19 vaccine and regular exercise practice, with a significant increase in IgA over time. However, there was a significant reduction in IgG after nine months of the 4th dose of the COVID-19 vaccine and regular exercise practice. The CG showed no differences in the levels of IgA and IgG over time. In the inter-group comparison (EG and CG), an increase in IgA and a decrease in IgG were observed in the EG after nine months of the 4th dose of the COVID-19 vaccine and regular exercise practice compared to the CG.

**Table 2:** Analysis of the immune response of the older adults between the groups: experimental group-physically active (EG) versus control group-insufficiently active (GC) after one and nine months of the fourth COVID-19 vaccine dose and regular exercise practice.

Immunological	Experimental Group (EG) Physically Active					Control Group (GC) Insufficiently Active					GE × GC (9 months)	Effect Size
	Baseline	1 month	9 months	*d*	p	Baseline	1 month	9 months	*d*	p^a^	p^b^	*d*^b^
IgA ‒ COVID-19 (nm)	1.7 ± 1.0	2.2 ± 0.8	2.6 ± 0.7	0.90	0.037^⁎#^	2.1 ± 1.2	2.0 ± 1.1	2.0 ± 0.9	0.83	0.980	0.019^b^	0.74
IgG ‒ COVID-19 (nm)	2.5 ± 1.1	2.7 ± 0.6	1.74±1.4	0.73	0.008^⁎#^	2.7 ± 1.0	2.8 ± 1.0	2.4 ± 0.8	0.41	0.258	0.036^b^	0.57

a MANOVA or MANCOVA test, intra-moments, with Bonferroni-adjusted post-hoc tests (*p* < 0.04 IgA and IgG) and the reported p-values remain interpretable against that threshold. Significant differences between moments: *before and after 1-month and ^#^before and after 9-months. Cohen’s *d*-test to verify the effect of the intervention (baseline × 9-months).

b Student *t*-test, independent, significant differences *p* < 0.05 and Cohen’s *d*-test to verify the effect of the intervention between-group comparisons at the 9-month time point.

**Fig. 3:**
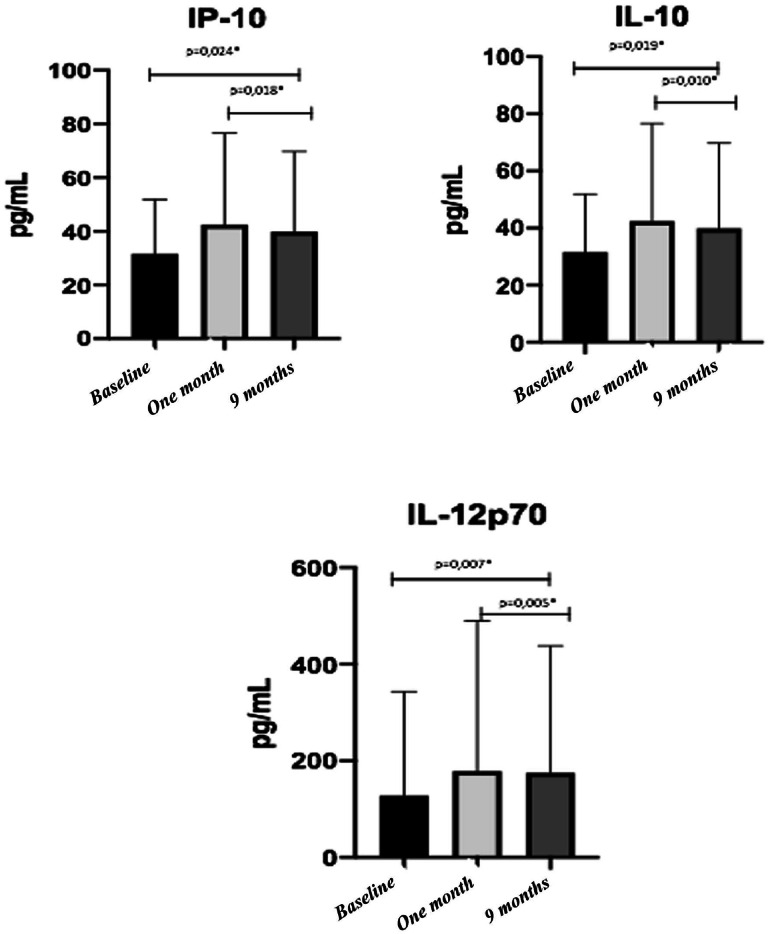
Systemic concentrations of IP-10, IL-10, and IL-12p70 in the older adults in Experimental Group ‒ Physically Active (GE) after one and nine months of the fourth COVID-19 vaccine dose and regular exercise practice.

Regarding the systemic inflammatory state, it can be observed in both Table 3 and Fig. 3 that the circulating levels of the cytokines IP-10, IL-10, and IL-12p70 increased significantly in the EG at both time points: after one month and nine months after the 4th dose of the COVID-19 vaccine and regular exercise practice, with a higher peak in the period one month after vaccination, when compared to the pre-vaccination period (baseline). Modulation of the immune-inflammatory response characterized by a balanced increase in both Th1-associated cytokines (IL-12p70, IP-10) and the regulatory cytokine IL-10, suggesting a controlled and effective immune activation following vaccination.

**Table 3:** Analysis of the systemic concentration of pro- and anti-inflammatory cytokines in the older adults between the groups: experimental group-physically active (EG) versus control group-insufficiently active (GC) after one and nine months of the fourth COVID-19 vaccine dose and regular exercise practice.

Inflammatory		Experimental Group (EG) Physically Active					Control Group (GC) Insufficiently Active				GE × GC (9-month)	Effect Size
	Baseline	1 month	9 months	*d*	p	d	1 month	9 months	*d*	p^a^	p^b^	*d*^b^
IL-6 (pg/mL)	5.9 ± 1.7	6.2 ± 1.9	5.8 ± 2.2	0.35	0.576	5.6 ± 1.3	6.4 ± 2.3	6.7 ± 2.6	0.12	0.824	0.331	0.37
IP-10 (pg/mL)	31.6 ± 20.2	42.4 ± 24.1	39.9 ± 29.8	0.52	0.020*#^&^	23.4 ± 15.4	39.6 ± 27.2	33.7 ± 22.7	0.53	0.188	0.040*	0.23
IL-8 (pg/mL)	16.2 ± 14.1	18.3 ± 10.2	19.7 ± 12.8	0.36	0.467	18.7 ± 9.0	24.4 ± 18.1	15.8 ± 8.5	0.33	0.358	0.522	0.35
IFN-α2 (pg/mL)	14.7 ± 21.0	21.4 ± 10.7	19.3 ± 10.2	0.27	0.402	9.1 ± 5.2	6.4 ± 2.3	7.3 ± 4.8	0.35	0.060	0.172	0.49
IFN-λ2/3 (pg/mL)	4.9 ± 2.8	5.2 ± 2.8	5.8 ± 2.1	0.36	0.369	3.8 ± 1.6	6.6 ± 2.5	4.2 ± 1.9	0.22	0.339	0.175	0.58
IL-10 (pg/mL)	15.4 ± 13.0	19.2 ± 12.8	17.6 ± 10.3	0.48	0.015*#^&^	16.0 ± 4.1	23.4 ± 4.7	15.6 ± 5.1	0.86	0.030*	0.016*	0.84
IL-1β (pg/mL)	20.9 ± 18.4	21.3 ± 13.2	24.0 ± 19.0	0.16	0.411	15.7 ± 10.2	20.2 ± 16.2	14.6 ± 8.2	0.41	0.034*	0.284	0.64
TNF-α (pg/mL)	50.0 ± 24.5	56.6 ± 33.4	66.3 ± 38.4	0.50	0.289	44.8 ± 28.7	32.6 ± 21.5	26.7 ± 12.6	0.81	0.425	0.090	0.98
IL-12p70 (pg/mL)	127.7 ± 21.5	178.7 ± 53.2	175.3 ± 62.2	0.96	0.004*#^&^	46.9 ± 33.2	65.9 ± 39.8	59.4 ± 32.5	0.38	0.228	0.122	0.95
IFN-λ1 (pg/mL)	5.5 ± 3.5	6.3 ± 4.6	5.9 ± 4.0	0.10	0.597	4.3 ± 2.6	6.5 ± 4.7	5.1 ± 3.3	0.26	0.408	0.526	0.21
IFN-β (pg/mL)	58.3 ± 12.2	58.0 ± 8.2	61.9 ± 10.2	0.32	0.217	86.0 ± 67.7	98.1 ± 68.6	76.4 ± 41.7	0.17	0.276	0.313	0.47
GM-CSF (pg/mL)	18.7 ± 10.5	23.4 ± 16.3	19.9 ± 15.6	0.90	0.194	9.6 ± 6.0	9.5 ± 6.7	9.8 ± 6.6	0.31	0.331	0.172	0.84
IFN-γ (pg/mL)	25.8 ± 15.9	29.5 ± 13.4	27.5 ± 14.1	0.12	0.143	15.8 ± 7.2	17.3 ± 8.4	22.1 ± 16.3	0.49	0.418	0.763	0.35

a MANOVA or MANCOVA test (*p* ≤ 0.05), intra-moments, with Bonferroni-adjusted post-hoc tests (*p* < 0.035, IP-10, IL-10, IL-1β, IL-12p70). Significant differences between moments: *Before and after 1-month.

# Before and after 9-months; and ^&^1-month and 9-months. Cohen’s *d*-test to verify the effect of the intervention (baseline × 9-months),.

b Student *t*-test, independent, significant differences *p* < 0.05 and Cohen’s *d*-test to verify the effect of the intervention between-group comparisons at the 9-month time point.

In the CG, a significant reduction was observed after nine months of the 4th dose of the COVID-19 vaccine and regular exercise practice in IL-10 (pg/mL) and IL-1β (pg/mL), with no differences for other variables. In the between-group comparison (EG vs. CG), the EG maintained higher IL-10 (pg/mL) concentrations after nine months of COVID-19 vaccination combined with regular exercise practice. Differences were also observed in Th1-associated markers, particularly IP-10 (Table 3). Overall, the EG exhibited an inflammatory profile characterized by higher IL-10 levels, changes in IL-12p70 and IP-10 concentrations, and improved pro-/anti-inflammatory cytokine ratios (e.g., IL-6/IL-10), suggesting a more regulated immune response (Table 3).

As shown in Table 4, the EG showed a significant reduction in the IL-6/IL-10 ratio after nine months of vaccination against COVID-19 and regular exercise practice when compared to the values observed in the pre-vaccination period, one month after vaccination against COVID-19 and regular exercise practice, and with CG. No significant differences were observed in the other variables.

**Table 4:** Analysis of inflammatory modulation (assessed through the ratio between cytokines with a pro-inflammatory profile and the anti-inflammatory cytokine IL-10) in the older adults between the groups: Experimental Group-physically active (EG) versus Control Group-insufficiently active (GC) after one and nine months of the fourth COVID-19 vaccine dose and regular exercise practice.

Inflammatory Modulation	Experimental Group (EG) Physically Active					Control Group - (GC) Insufficiently Active					GE × GC (9 months)	Effect size
	Baseline	1 month	9 months	*d*	p	Baseline	1 month	9 months	*d*	p^a^	p^b^	*d*^b^
IL-6 / IL 10 (pg/mL)	0.9 ± 0.4	0.9 ± 0.4	0.7 ± 0.3	0.56	0.007#^&^	1.2 ± 0.4	1.2 ± 0.4	1.3 ± 0.6	0.20	0.352	0.020^b^	0.99
IL-8/IL-10 (pg/mL)	1.8 ± 1.0	2.0 ± 1.1	1.9 ± 0.8	0.11	0.762	3.8 ± 2.1	4.6 ± 2.7	3.3 ± 2.0	0.24	0.449	0.030^b^	0.91
TNF-α/IL-10 (pg/mL)	6.2 ± 3.0	4.8 ± 2.8	4.8 ± 2.9	0.44	0.467	5.3 ± 3.3	6.5 ± 4.3	4.9 ± 3.5	0.11	0.381	0.994	0.08
IL-12p70/IL-10 (pg/mL)	6.5 ± 5.2	6.6 ± 5.3	9.9 ± 6.2	0.59	0.341	5.3 ± 3.7	7.8 ± 5.8	6.7 ± 5.5	0.29	0.399	0.366	0.54
IL-1β/IL-10 (pg/mL)	2.4 ± 1.2	2.0 ± 0.9	2.0 ± 1.0	0.36	0.259	2.6 ± 0.5	2.8 ± 1.0	2.8 ± 0.8	0.30	0.469	0.058	0.68
IP-10/IL-10 (pg/mL)	4.7 ± 3.3	6.3 ± 4.6	5.8 ± 4.4	0.28	0.230	5.1 ± 3.8	7.8 ± 5.0	7.3 ± 5.6	0.45	0.889	0.499	0.29
IFN-γ/IL-10 (pg/mL)	2.7 ± 1.1	2.6 ± 1.2	2.5 ± 1.1	0.18	0.576	3.1 ± 0.7	3.2 ± 1.0	4.5 ± 2.7	0.70	0.962	0.170	0.87
IFN-α2/IL-10 (pg/mL)	1.1 ± 0.8	1.2 ± 0.9	0.9 ± 0.7	0.26	0.232	0.9 ± 0.6	0.8 ± 0.6	0.8 ± 0.7	0.15	0.359	0.813	0.14
IFN-β/IL-10 (pg/mL)	10.1 ± 6.1	9.3 ± 5.1	9.7 ± 5.5	0.06	0.863	19.6 ± 9.1	22.1 ± 9.5	16.5 ± 10.7	0.32	0.110	0.078	0.79
IFN-λ1/IL-10 (pg/mL)	0.6 ± 0.3	0.7 ± 0.3	0.7 ± 0.3	0.33	0.868	0.8 ± 0.4	1.0 ± 0.5	0.9 ± 0.4	0.25	0.118	0.124	0.46
IFN-λ2/IL-10 (pg/mL)	0.6 ± 0.3	0.6 ± 0.3	0.6 ± 0.2	0.01	0.820	0.8 ± 0.4	0.9 ± 0.3	0.8 ± 0.4	0.01	0.990	0.115	0.23
GM-CSF/IL-10 (pg/mL)	1.4 ± 0.4	1.4 ± 0.4	1.5 ± 0.4	0.25	0.838	1.7 ± 0.7	1.8 ± 0.9	1.7 ± 0.9	0.01	0.968	0.490	0.28

a MANOVA (*p* < 0,05) or MANCOVA test, intra-moments, with Bonferroni-adjusted post-hoc tests (*p* < 0.035, for IL-6/IL 10 and IL-8/IL-10). Significant differences between moments: ^†^Before and after 1-month.

# Before and after 9-months; and ^&^1-month and 9-months. Cohen’s *d*-test to verify the effect of the intervention (baseline × 9-months).

b Student *t*-test, independent, significant differences *p* < 0.05 and Cohen’s *d*-test to verify the effect of the intervention between-group comparisons at the 9-month time point.

In the CG, no significant differences were observed for any variable across the different time points of monitoring. When comparing the groups (EG and CG non-regular exercise), a better balance in the modulation of IL-6/IL-10 (pg/mL) and IL-8/IL-10 (pg/mL) was observed in the EG after nine months of vaccination against COVID-19 and regular exercise practice compared to the CG, with no differences in the other modulation indices (Table 4).

**Fig. 4:**
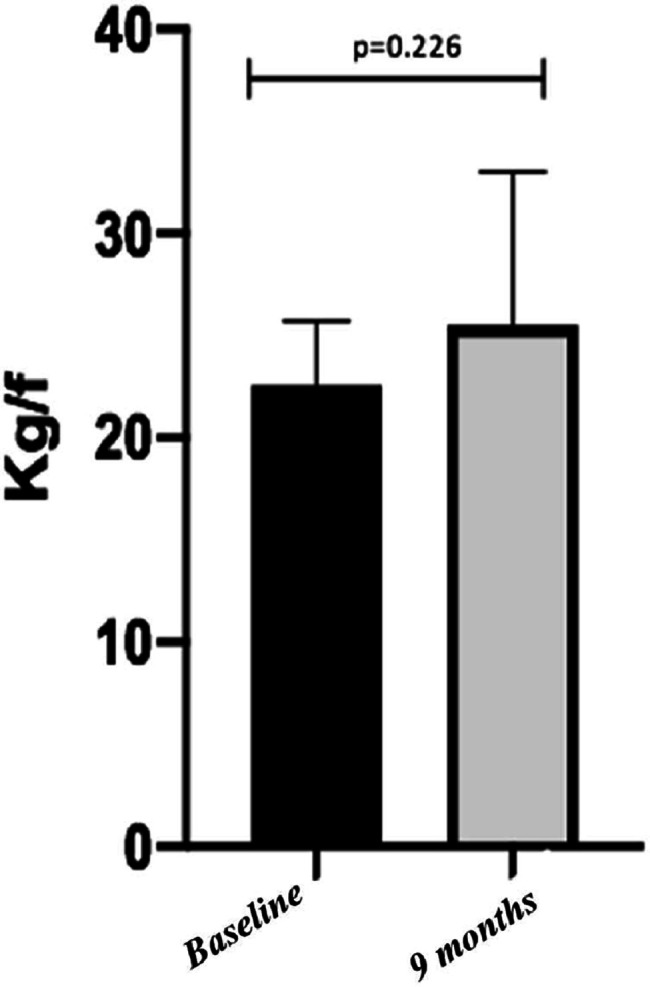
No significant changes in handgrip strength were observed in either group (GE and CG) over the nine-month period of the fourth COVID-19 vaccine dose and regular exercise practice.

The GE showed significant improvements in functionality, evidenced by the shorter time required to complete the Step test and Floor transfer test, as well as improvements in gait through the walking test and physical activity levels as assessed by the modified Baecke questionnaire, one and nine months after COVID-19 vaccination and regular exercise practice, when compared to the pre-vaccination period. However, functional performance as assessed by the TUG test and fall risk as measured by the FRAQ questionnaire did not show significant differences when comparing pre-vaccination and one month after vaccination and regular exercise practice (Table 5). Regarding manual grip strength, no statistical differences were observed during the evaluated period (Fig. 4). In the CG, no significant differences were observed for any variable across the different time points of monitoring. When comparing the groups (GE and CG), it was observed that after nine months of the 4th dose of the COVID-19 vaccine and regular exercise practice, the GE showed better physical performance, reflected by reduced time in the physical tests (step test and floor transfer test), as well as a reduction of the walking test and an increased level of physical activity compared to the CG.

**Table 5:** Comparison of functional aspects, risk of falls, and walking older adults between the groups: Experimental Group-physically active (EG) versus Control Group-insufficiently active (GC) after one and nine months of the fourth COVID-19 vaccine dose and regular exercise practice.

Physical-Functional Aspects	Experimental Group (EG) Physically Active				Control Group - (GC) Insufficiently Active				GE × GC (9 months)	Effect size
	Baseline	9 months	*d*	p	Baseline	9 months	*d*	p^a^	p^b^	*d*^b^
Step Test (time/min.)	9.7 ± 0.6	8.0 ± 2.2	0.43	0.026^a^	10.3 ± 1.8	10.8 ± 1.9	0.27	0.570	0.011^a^	0.98
Floor Transfer (seconds/s)	7.0 ± 2.9	5.4 ± 2.3	0.61	0.009^a^	6.3 ± 3.6	6.7 ± 3.0	0.12	0.576	0.022^a^	0.48
Timed Up and Go ‒ TUG (time/s)	7.6 ± 1.3	6.1 ± 1.7	0.90	0.098	7.4 ± 1.8	7.0 ± 1.7	0.14	0.987	0.077	0.52
Walking Test ‒ 6MWT (time/min.)	6.0 ± 0.8	5.0 ± 1.1	0.93	0.022^a^	6.0 ± 1.1	6.8 ± 1.0	0.07	0.322	0.015^a^	0.99
Fall risk awareness questionnaire ‒ FRAQ (score)	18.6 ± 5.1	19.4 ± 2.6	0.19	0.629	21.5 ± 3.0	20.3 ± 2.8	0.16	0.152	0.330	0.34
Baecke Questionnaire (score)	15.2 ± 6.0	21.5 ± 5.2	1.0	0.007^a^	15.9 ± 4.7	16.2 ± 4.9	0.18	0.100	0.025^a^	0.98

a Student *t*-test, dependent, significant differences *p* < 0.05. Cohen’s *d*-test to verify the effect of the intervention (baseline × 9-months).

b Student *t*-test, independent, significant differences *p* < 0.05 and Cohen’s *d*-test to verify the effect of the intervention between-group comparisons at the 9-month time point.

The EG showed significant improvements in plantar support, with a reduction in plantar load (peak pressure and maximum force) on the forefoot and an increase in midfoot support compared before and after nine months of the 4th dose of the COVID-19 vaccine and regular exercise practice, promoting better flattening of the plantar support for improved distribution of the force load during walking. However, this support was still insufficient to reduce plantar load (peak pressure and maximum force) on the medial and lateral rearfoot, as well as the contact area in different regions of the feet (Table 6). In the CG, no significant differences were observed for any variable across the different time points of monitoring. When comparing the groups (GE and CG) after nine months of the 4th dose of the COVID-19 vaccine and regular exercise practice, it was observed that the GE showed a reduction in plantar load (peak pressure and maximum force) on the midfoot and rearfoot (medial and lateral) compared to the CG, with no differences in the contact area across the different regions of the feet.

**Table 6:** Comparison of the biomechanical parameters of gait in older adults between the groups: Experimental Group-physically active (EG) versus Control Group-insufficiently active (GC) after one and nine months of the fourth COVID-19 vaccine dose and regular exercise practice.

Variables	Foot Regions	Experimental Group (EG) Physically Active				Control Group (GC) Insufficiently Active				GE × GC (9 months)	Effect size
		Baseline	9 months	*d*	p	Baseline	9 months	*d*	p^a^	p^b^	*d*^b^
Contact Area (cm^2^)	Forefoot	10.0 ± 1.2	9.8 ± 1.3	0.15	0.753	9.4 ± 2.4	10.5 ± 2.5	0.10	0.068	0.462	0.35
	Midfoot	13.2 ± 9.8	10.2 ± 11.6	0.27	0.509	11.6 ± 9.5	15.6 ± 10.7	0.12	0.164	0.282	0.48
	Medial rearfoot	18.6 ± 2.5	19.5 ± 2.8	0.33	0.406	20.3 ± 2.7	20.9 ± 2.7	0.09	0.425	0.353	0.50
	Lateral rearfoot	18.7 ± 2.1	19.3 ± 3.0	0.23	0.647	20.5 ± 2.6	21.0 ± 2.8	0.10	0.520	0.244	0.58
Peak Pressure (KPa)	Forefoot	349.9 ± 56.3	330.8 ± 49.7	0.35	0.005^a^	276.1 ± 98.5	254.0 ± 92.8	0.23	0.492	0.891	0.62
	Midfoot	127.4 ± 74.5	135.1 ± 94.0	0.19	0.002^a^	163.9 ± 84.2	163.4 ± 79.0	0.07	0.784	0.037^a^	0.33
	Medial rearfoot	340.0 ± 88.7	344.0 ± 64.3	0.15	0.010^a^	352.7 ± 57.6	358.4 ± 54.1	0.10	0.242	0.012^a^	0.42
	Lateral rearfoot	323.7 ± 91.9	335.8 ± 77.8	0.14	0.009^a^	380.6 ± 73.5	373.7 ± 76.7	0.14	0.143	0.018^a^	0.50
Maximum force (N/BW)	Forefoot	15.5 ± 4.3	14.3 ± 3.2	0.31	0.011^a^	10.3 ± 5.2	10.6 ± 5.5	0.09	0.841	0.191	0.82
	Midfoot	8.0 ± 3.5	9.2 ± 5.2	0.27	0.007^a^	13.0 ± 3.2	14.2 ± 3.4	0.10	0.330	0.021^a^	0.98
	Medial rearfoot	31.0 ± 9.3	35.2 ± 11.7	0.39	0.005^a^	40.8 ± 10.9	41.5 ± 11.3	0.06	0.667	0.025^a^	0.54
	Lateral rearfoot	29.3 ± 8.6	33.7 ± 12.0	0.42	0.006^a^	40.3 ± 13.6	39.8 ± 13.3	0.07	0.803	0.011^a^	0.48

a Student *t*-test, dependent, significant differences *p* < 0.05. Cohen’s *d*-test to verify the effect of the intervention (baseline × 9-months).

b Student *t*-test, independent, significant differences *p* < 0.05 and Cohen’s *d*-test to verify the effect of the intervention between-group comparisons at the 9-month time point.

## Discussion

The aim of the current study was to compare immune response, inflammation, physical-functional performance, and gait at one and nine months after the fourth dose of the COVID-19 vaccine between physically active and insufficiently active older adults. Based on this theme, the main results showed that the GE (physically active) exhibited a distinct modulation of the antibody response to the virus, characterized by increased IgA levels and a reduction in IgG over time, compared with the CG (insufficiently active). Together, these findings suggest a differential trajectory of the immune response following vaccination against COVID-19 in individuals engaging in regular physical activity; however, the clinical significance of these between-group differences remains uncertain. In addition, a modulation of the immune-inflammatory profile was observed, marked by a balanced increase in both Th1-associated cytokines (IL-12p70, IP-10) and the regulatory cytokine IL-10, suggesting a controlled and effective immune activation following vaccination against COVID-19 and regular exercise practice compared with CG (insufficiently active). In inflammatory modulation, differences can be observed in the IL-6/IL 10 ratio (pg/mL), with a reduction nine months after vaccination and regular exercise practice when compared to the period before and one month after vaccination. The observed differences are likely explained by long-term engagement in regular physical exercise rather than by the fourth vaccine dose. Due to the observational cohort design of the study, comparing older physically active vs. insufficiently active, causal relationships cannot be established, and the results should be interpreted as associations between habitual regular exercise behavior and post-fourth-dose immunological, inflammatory, functional, and biomechanical outcomes.

In the literature, the search for adjuvants to improve vaccine response and protection against infections is evident, with physical exercise being considered as a behavioral intervention capable of improving immunological and inflammatory responses 46, 47, 48, 49, thus, potentially serving as an adjuvant to modulate the protective immunological response in response to vaccination against COVID-19 .^47^ Scientific evidence suggests that regular physical exercise can act as a natural adjuvant in modulating immune and inflammatory responses to vaccination ‒ particularly to the COVID-19 vaccine ‒ including in older adults and individuals with autoimmune rheumatic diseases. This hypothesis is supported by several studies ^50^^,^^51^, showing that regular exercisers develop stronger antibody responses after vaccination in physically active older adults (EG) compared with insufficiently active older adults (CG). The presented findings confirm that continuous exercise plays a crucial role in modulating the immune response to COVID-19 vaccination. Only volunteers who maintained a regular chronic exercise routine exhibited a significant increase in systemic IgA and IgG levels against SARS-CoV-2 antigens one month after vaccination, while the CG, composed of insufficiently active older adults, showed no such improvement. This difference is not solely attributed to immunosenescence (the natural age-related decline in immune function) but reinforces that an active lifestyle can mitigate its adverse effects and prolong vaccine efficacy in older adults. However, these immunological modulations should not be interpreted as direct indicators of protection against infection. Over time, several studies have reported a decline in circulating IgG levels after vaccination ,^52^, ^53^, ^54^ a finding also confirmed in this study. However, an interesting observation was the unexpected increase in IgA levels nine months after vaccination among the Exercise Group (EG), in contrast to the Control Group (CG), whose IgA levels remained stable or decreased. This result contradicts previous studies reporting a natural decline in IgA over time following vaccination .^55^^,^^56^

This IgA increase could reflect recent, asymptomatic exposure to SARS-CoV-2, since participants trained together, but it also highlights the ability of regular exercise to sustain long-term immune modulation. It is worth noting that IgA antibodies, primarily found in mucosal tissues, are essential for initial immune defense against SARS-CoV-2 and are associated with a lower risk of infection .^57^ Beyond antibody production, the study also assessed participants’ systemic inflammatory profile. One month after vaccination, physically active individuals showed increased levels of pro-inflammatory cytokines IP-10 and IL-12p70, as well as the anti-inflammatory cytokine IL-10. IP-10 should be interpreted cautiously as a marker of immune activation rather than immune improvement, reflecting interferon-driven signaling and recruitment of immune cells .^58^ IL-12p70 is a key Th1 cytokine involved in B-cell activation and antibody production, thus strengthening vaccine responses .^59^ IL-10 exerts a regulatory anti-inflammatory effect, preventing excessive inflammation and maintaining immune balance .^60^ Thus, these cytokine changes indicate immune activation with a regulatory balance in defense against SARS-CoV-2.

The findings of the present study indicate that continuous exercise is associated with modulation of the immune-inflammatory response, characterized by a balanced increase in both Th1-associated cytokines (IL-12p70, IP-10) and the regulatory cytokine IL-10. This pattern suggests a controlled and effective immune activation following vaccination (against COVID-19) in older adults who engage in regular exercise, compared with the control group (insufficiently active). This regulation was further reflected in the balanced IL-6/IL-8 to IL-10 ratios, indicating a well-regulated inflammatory state .^61^ In contrast, the Control Group (CG ‒ insufficiently active older adults) exhibited a reduction in IL-1β and IL-10 levels nine months after vaccination, which may explain their weaker immune response, since other studies reported an increase in these cytokines after booster doses .^62^ Importantly, these antibody changes represent immunological responses and not established clinical correlates of protection, such as reduced infection or disease severity. Overall, the data demonstrate that regular and continuous physical exercise enhances both immune and inflammatory responses to COVID-19 vaccination in older adults, contributing to immune activation, inflammation control, and attenuation of immunosenescence effects. However, the clinical significance of these immunological changes, including protection from infection or disease severity, cannot be determined within the scope of this study.

Studies have revealed that moderate to vigorous physical exercise is able to improve immune function, which is reflected in greater antibody or cell-mediated responses to vaccination .^46^, ^47^, ^48^, ^49^^,^^63^ According to Barni et al ^48^, in a recent systematic review with meta-analysis, physical training proved to be effective in increasing vaccine antibodies, both in adults and older adults, with a significant increase in direct responses to the vaccine, and the quantities of IL-6 and leukocytes in individuals who underwent physical exercises in relation to the control (CG sedentary).

Aging is characterized by several changes, including exacerbated inflammatory responses mediated by the innate immune system with reduced capacity to protect against infections, leading to more severe consequences from bacterial and viral infections and a reduced response to vaccination .^64^ This pro-inflammatory status renders older individuals susceptible to tissue-damaging immunity and chronic inflammatory diseases. The regular practice of regular chronic exercise at a moderate intensity (64%–76% of the maximal heart rate)^65^ induces the activation of several signaling pathways, conveying a sustained anti-inflammatory and antioxidant response. Some of the positive effects of chronic exercise on the immune system are related to increased T-cell proliferative capacity, neutrophil function, and cytotoxic activity of NK cells .^66^ According to Amatriain-Fernández et al. (2020) ^67^, chronic exercise of moderate intensity can increase T-cell proliferative capacity and differentiation into an activated phenotype, enhance immune cell phagocytic and cytotoxic activity, increase production of anti-inflammatory mediators, elevate levels of antioxidant low-molecular-weight compounds and enzyme activities, and boost cell energy production.

In the present study, an increase in the immune response to the COVID-19 virus can be observed in EG (physically active older adults) through improvement in the immunological and inflammatory response of the older adults, one and nine months after the 4th dose of vaccination against COVID-19 and regular exercise practice. These points aid understanding of the effectiveness of the 4th dose of the vaccine associated with regular chronic exercise to maintain the antibody response and inflammatory markers, favoring the balance between pro-inflammatory and anti-inflammatory cytokines, which can be evidenced through the inflammatory modulation by the IL-6/IL 10 ratio (pg/mL), which remained reduced 9-months after vaccination and regular exercise practice when compared to the period before and one month after vaccination against COVID-19. For Paula et al^68^ and Sá Filho et al ^69^, studies have shown significant findings in favor of High Intensity Interval Training (HIIT) protocols when compared to moderate intensity exercise, showing how the immunological system responds to vigorous high intensity training. However, in this study, the intervention protocol was of moderate intensity, and the results also showed an increase in the immunological response, as well as in the functional response, after the 4th dose of the COVID-19 vaccine, when compared to the control group.

According to the literature, older adults are a risk group for COVID-19 due the increase in inflammatory cells, such as cytokines and interleukins, typical of the aging process, which can have a major impact from COVID-19, and physical exercise can modulate the response of several inflammatory mediators that favor disease control .^37^^,^^46^, ^47^, ^48^, ^49^^,^^63^ According to Fernández-Lázaro et al^70^ physical exercise exerts immunomodulatory effects, controls the viral entry point, modulates inflammation, stimulates nitric oxide synthesis pathways, and establishes control over oxidative stress. These points were corroborated by the results of the current study, which evaluated the effects of regular exercise practice after the 4th dose of vaccination against COVID-19 for older adults (physically active versus insufficiently active) over one and nine months of monitoring. In addition, the mental and physical effects of repeated lockdowns during COVID-19 have cumulative negative effects on interpersonal interaction and loneliness .^71^ Another study involving adolescents suggests an association between physical activity, boredom, and the quarantine experience. Adolescents who were more physically active, particularly with high-intensity exercise, reported feeling less bored and exhibited lower levels of fear regarding COVID-19. Although this study focused on older adults, it is reasonable to suggest that these mental and physical conditions were common across age groups during the COVID-19 pandemic .^72^

Another important finding observed in the present study was the increase in functionality, with less time taken to perform physical performance tests, as well as the six-minute walk test and increased practice of physical activity over the 9-months after vaccination against COVID-19 and regular exercise practice. Most of the studies in the literature on the functional behavior of older people were aimed at verifying the benefits of a physical exercise program after hospitalization for COVID-19, and the results showed an increase in handgrip strength, gait speed, lower limb strength, balance, and fragility .^73^, ^74^, ^75^

According to Miller et al^74^ twelve months of aerobic exercise combined with resistance exercise improved gait and flexibility for functional activities. In the study by Chen et al^75^ physical exercises led to better physical function and mental health outcomes in older adults during the COVID-19 pandemic. In the current study, it was also possible to observe not only functional improvement, but also improvement in gait parameters with a reduction in plantar load (forefoot) and an increase in the support of the midfoot, favoring good flattening of the plantar support for better distribution of the load during walking 9-months after the 4th dose of vaccination against COVID-19 and regular exercise practice. The clinical relevance of this study was to understand the immunological, inflammatory, functional, and gait response over one and nine months of monitoring after the 4th dose of vaccination against COVID-19 and regular exercise practice.

The present findings help to understand the immunological, inflammatory, functional, and gait response over one and nine months of monitoring after the 4th dose of vaccination against COVID-19 and regular exercise practice in older adults (physically active and insufficiently active). These findings can be used for research purposes and to guide the conservative treatment of older people with knee osteoarthritis who returned to exercise after a period of social isolation resulting from the COVID-19 pandemic and the benefits of the 4th dose of the vaccine combined with regular exercise practice for a better response to physical performance-functional. Some scientific evidence has shown that moderate to vigorous physical exercise is capable of improving immune function, which is reflected in greater antibody or cell-mediated responses to vaccination .^46^, ^47^, ^48^, ^49^, ^50^ Thus, in this study, the authors were able to monitor the older adults over nine months after 4-doses of vaccination and regular exercise practice, and the authors were able to notice an improvement in immunity, in inflammatory modulation, and in the functional and biomechanical performance of load rates during walking, especially in physically active older adults. However, future perspectives with longer monitoring of older women who practice physical exercise are necessary to recommend effective intervention strategies to prevent functional limitations and the risk of falls while walking.

The main limitation of this study was the small sample size, which may limit the generalizability of the findings to a larger and more diverse population. Despite careful sample selection and study cohort over 9-months of monitoring, the results can strongly be considered hypothesis-generating due to the sample size and the multiple outcomes assessed, and should be validated in future studies with larger cohorts and increased sample size. In addition, the Bonferroni correction was applied to control for multiple comparisons and minimize the risk of Type I error. Thus, the results were interpreted and discussed with appropriate caution because the study cannot disentangle the effects of long-term habitual exercise from the acute effects of exercise resumption following vaccination. The exclusion of other factors that could affect immune and inflammatory responses (such as the use of immunosuppressive medications or the presence of other inflammatory diseases) may limit the applicability of the results to more heterogeneous populations.

Future research would benefit from expanding the sample size to include a broader range of demographic characteristics, such as different age groups, comorbidities (e.g., cardiovascular diseases, diabetes), and immunological status (individuals with autoimmune diseases or those using immunosuppressive medications). This would allow for a more accurate analysis of how these factors influence the immune response to exercise and vaccination. Moreover, investigating different exercise modalities, such as aerobic activities, strength training, flexibility exercises, or resistance training, would be valuable to assess which type of exercise has the greatest impact on the immune response post-vaccination. Comparing different exercise intensities could also reveal the thresholds of physical overload in older adults and their effect on modulating the immune response. The issue of optimal exercise dose also requires further exploration. Future studies could assess not only the quantity and frequency of exercise but also the impact of varying intensity levels (e.g., interval training vs. continuous exercise) on optimizing the immune response. This aspect is particularly relevant in aging contexts, where the risk of physical overload is a concern, and personalizing exercise protocols could ensure better outcomes for individuals.

Another promising direction would be to explore combined interventions, such as a multimodal approach that integrates exercise with nutritional strategies (such as the intake of micronutrients, antioxidants, or omega-3 fatty acids) and supplementation, as well as investigating the impact of different diets on the vaccine response. Furthermore, it could be valuable to conduct studies that combine exercise with other complementary therapies (such as stress reduction techniques or psychological interventions) to evaluate the influence of overall well-being on immunity.

## Conclusion

The practice of regular physical exercise was associated with more favorable immune, inflammatory, physical-functional, and gait outcomes at one and nine months after the fourth dose of the COVID-19 vaccine in physically active older adults compared with insufficiently active peers. These findings suggest that long-term regular physical exercise is associated with a more robust and balanced immune-inflammatory profile characterized by a balanced increase in both Th1-associated cytokines (IL-12p70, IP-10) and the regulatory cytokine IL-10, suggesting a controlled and effective immune activation following vaccination and better physical-functional performance with reduced plantar overloading during gait, particularly among physically active older adults. Given that this study was conducted in a specific cohort, the results should be interpreted as associative rather than causal and should not be generalized as definitive clinical recommendations. Nevertheless, the findings support the potential relevance of structured regular physical exercise programs as a rehabilitation strategy to promote functional and immunological resilience in aging populations.

## Ethical approval

All procedures conducted in this study involving human participants adhered to the ethical standards of the institutional and/or national research committee, in accordance with the Helsinki Declaration. The study received approval from the Research Ethics Committee of the Universidade Santo Amaro-UNISA (Approval n°5.418.231).

## Data availability

Indication that the data must be requested from the corresponding author (APR on e-mail: apribeiro@alumni.usp.br).

## Authors’ contributions

The corresponding author is responsible for ensuring that the descriptions are accurate and agreed upon by all authors.

## Funding

This study did not receive financial support for the research.

## Declaration of competing interest

The authors declare no conflicts of interest.

## Appendix Supplementary materials

Image, application 1
