# Supplementary material for: Immunomodulatory, inflammatory, functional and biomechanical benefits of chronic exercise in physically active vs. insufficiently active older after fourth COVID-19 vaccine dose: nine months cohort
Source: Clinics (Sao Paulo). 2026 Jun 26;81:101014. doi: 10.1016/j.clinsp.2026.101014 (PMC13319388; doi:10.1016/j.clinsp.2026.101014)
Supplement: Supplementary file 1 [file mmc1.docx]

**CLINICS-D-25-01587_Supplementary Material**

**Supplementary Table S1** Description of the supervised exercise training program (FITT-Multicomponent exercises).

| **FITT-Multicomponent exercises** | **Description ‒ Exercise Training Program** |
| --- | --- |
| Frequency | 3 sessions per week |
| Intensity | ***Aerobic training:*** Moderate intensity, 60–75% of maximum heart rate (HRmax), calculated using the equations 220 − age and 208 − 0.7 × age. Heart rate was monitored using a Polar FT1 heart rate monitor (Polar, Finland). ***Resistance training*:** Moderate intensity, 50%–60% of one-repetition maximum (1RM). |
| Time (Duration) | ***Aerobic component:*** 30 minutes per session. ***Resistance component***: 30 minutes per session. Total session duration: Approximately 60 minutes. |
| Type | ***Multicomponente training protocol:***  1) Aerobic exercises performed continuously at moderate intensity;  2) Localized resistance exercises targeting upper and lower limbs: abdominal muscles, gluteal muscles, and postural stabilization muscles (including dorsal and lumbar regions). |
| Resistance Training Structure | **5–10 exercises per session**; 2 sets of 10–20 repetitions per exercise, performed using free weights or weight plates. |
| Program Supervision | All sessions were supervised by the same qualified ***physical education professional***, who monitored exercise execution, intensity, and progression. |
| Progression | Exercise intensity and workload were progressively adjusted throughout the program according to individual tolerance and ***ACSM/AHA recommendations.*** |
| Adherence Monitoring | Attendance was recorded for all sessions. Only participants with ≥80% attendance across the supervised sessions were included in the final analysis. |
| Training Setting | Centro Educacional e Esportivo Ibirapuera (SEME), São Paulo, Brazil. |
